# MiR145-5p inhibits proliferation of PMVECs via PAI-1 in experimental hepatopulmonary syndrome rat pulmonary microvascular hyperplasia

**DOI:** 10.1242/bio.044800

**Published:** 2019-11-04

**Authors:** Yang Chen, Congwen Yang, Yujie Li, Lin Chen, Yong Yang, Karine Belguise, Xiaobo Wang, Kaizhi Lu, Bin Yi

**Affiliations:** 1 Department of Anesthesia, Southwest Hospital, Third Military Medical University (Army Medical University), Chongqing 400038, China; 2 Laboratoire de Biologie Cellulaire et Moléculaire du Contrôle de la Prolifération (LBCMCP), Centre de Biologie Intégrative (CBI), Université de Toulouse, CNRS, Université Paul Sabatier (UPS), 31062 Toulouse, France

**Keywords:** Hepatopulmonary syndrome (HPS), Plasminogen activator inhibitor-1 (PAI-1), MiR145-5p, Pulmonary microvascular endothelial cells (PMVECs)

## Abstract

Hepatopulmonary syndrome (HPS) is a triad of advanced liver disease, intrapulmonary vasodilatation and arterial hypoxemia. Increasing evidence shows that HPS is associated with pulmonary microvascular hyperplasia. The aim of this work was to investigate the underlying mechanism of miR-145 in regulating the proliferation of pulmonary microvascular endothelial cells (PMVECs) and angiogenesis in HPS via plasminogen activator inhibitor-1 (PAI-1). To test this, morphology score and number of pulmonary microvascular were assessed in lung tissues from rats with HPS by Hematoxylin and Eosin (H&E) staining. Expression levels of PAI-1 were assessed in lung tissues from HPS rats, as well as in PMVECs treated with HPS rat serum. We also selected the putative microRNA binding site on PAI-1 by bioinformatics analysis. Then, miR145-3p and miR145-5p expression levels in the lungs and PMVECs of rats were detected by qRT-PCR because miR145-5p is a microRNA binding site on PAI-1. In addition, the effects of miR-145-5p regulation on PAI-1 were examined by upregulation and downregulation of miR-145-5p and specific lentivirus transfection was used to overexpress and knockdown PAI-1 to assess PAI-1 function on PMVECs proliferation. Our data showed that levels of PAI-1 expression in lung tissue of rats increased significantly when rats were treated with common bile duct ligation. We found that levels of miR-145-5p were frequently downregulated in HPS tissues and cell lines, and overexpression of miR-145-5p dramatically inhibited PMVECs proliferation. We further verified PAI-1 as a novel and direct target of miR-145-5p in HPS. MiR-145-5p inhibits PAI-1 synthesis and the expression changes of PAI-1 directly affect the proliferation of PMVECs. We concluded that miR-145-5p negatively regulates PMVEC proliferation through PAI-1 expression. In addition, overexpression of miR-145-5p may prove beneficial as a therapeutic strategy for HPS treatment.

## Abstract (precis)

**Summary:** Our findings provide proof of principle that microRNAs may be useful for the future development of novel therapeutic strategies in HPS.

## INTRODUCTION

Hepatopulmonary syndrome (HPS) is a refractory disease with high morbidity and mortality characterized by advanced liver disease, hypoxemia and intrapulmonary vascular dilatation (IPVD) (Kammoun et al., 2007). Multiple growth factors and circulating cytokines are released by a diseased liver and contribute to the occurrence of IPVD, leading to further development of HPS (Miyamoto et al., 2010). Multiple cytokines released by a diseased liver result in the activation and upregulation of signal proteins from multiple signaling pathways, such as TGF-β/Smad, Annexin A1/A2, PI3K-Akt and PKC/ERK, leading to myogenic differentiation and proliferation of pulmonary microvascular endothelial cells (PMVECs) (Liu et al., 2017, 2015; Yi et al., 2013). These changes are the main pathophysiological changes that occur in IPVD and result in pulmonary microvascular pathological hyperplasia and expansion (He et al., 2017).

It is widely accepted that angiogenesis is the basis of capillary dilation and arteriovenous malformation. However, the mechanism of alveolar capillary angiogenesis in HPS is incompletely understood.

Plasminogen activator inhibitor-1 (PAI-1), also known as endothelial plasminogen activator inhibitor or serpin E1, is a protein that in humans is encoded by the SERPINE1 gene (de Faria et al., 2019). PAI-1 is a serine protease inhibitor that functions as the principal inhibitor of urokinase (uPA) and tissue plasminogen activator (tPA), activators of fibrinolysis and plasminogen (Jevric et al., 2019). The other PAI, PAI-2, is secreted by the placenta and only present during pregnancy. In addition, PAI-1 is the main inhibitor of the plasminogen activators (Jin et al., 2019), and elevated PAI-1 is a risk factor for thrombosis and atherosclerosis. It has been reported that inhibition of PAI-1 and TGF-β1/Smad signaling leads to suppression of human colorectal carcinoma cell proliferation and migration (Wang et al., 2017). A number of studies have reported that the viability and migratory ability of human umbilical vein endothelial cells (HUVECs) is regulated by PAI-1(Patel et al., 2011). PAI-1 acts as a key regulator of angiogenesis via its effects on the degradation of extracellular matrix and the adhesion function of cells. PAI-1 also regulates pulmonary artery smooth muscle cell (PASMC) phenotypes (Chen et al., 2018). Previous studies have demonstrated that expression of PASMC phenotype marker proteins is the primary pathophysiological change in PMVECs myogenic differentiation (Yi et al., 2015). PAI-1 is also elevated in acute lung injury, which is characterized by a loss of endothelial barrier function and upregulation of vascular permeability (Makarova et al., 2011). It is important to identify the adjustment function of PAI-1 on pulmonary microvascular hyperplasia, which may be a key upstream target for IPVD treatment and provide a new approach for HPS prevention.

MicroRNAs (miRNAs) are endogenous noncoding RNAs from 20 to 24 nucleotides in length that regulate one-third of human gene expression in many biological processes, including development, cell proliferation, cell differentiation and tumorigenesis (Bartel, 2009; Shukla et al., 2011). In general, miRNAs post-transcriptionally regulate expression of target genes by specifically binding and cleaving mRNAs or inhibiting their translation (Ivey and Srivastava, 2015). In animals, most investigated miRNAs repress gene expression by incompletely binding to the 3′-untranslated region (UTR) of target mRNAs (Rajasekaran et al., 2016). It has been reported that miRNA association results in translational repression, frequently accompanied by a considerable degradation of mRNAs by a non-RNAi mechanism (Filipowicz et al., 2008). Previous studies and our initial research have demonstrated that several miRNAs have roles in regulating the pathology of HPS, including miR-206, miR-199a-5p and miR-9 (Chen et al., 2014; Gao et al., 2015; Xu et al., 2015). To date, these findings are related to miRNAs regulating PASMC phenotypic modulation and proliferation. However, close attention should be given to the regulation of angiogenesis in HPS and PMVEC proliferation by microRNA. A number of studies have demonstrated that PAI-1 is regulated by different miRNAs and plays a regulatory role in various biological processes in many diseases (Lin et al., 2017; Zhang et al., 2018). Here, we hypothesized that some miRNAs may regulate the PAI-1 signaling pathway to control the pathogenesis of HPS-associated IPVD.

The purpose of this work was to examine the role of microRNAs in regulating the proliferation of PMVECs and angiogenesis in HPS by PAI-1. We established an HPS model, detected expression of PAI-1 in lungs and PMVECs of rats, and found increased numbers of pulmonary microvascular in lung tissue of an experimental HPS rat model. Our data showed that expression of PAI-1 in lung tissue of rats increased significantly, while the lung injury score was noticeably upregulated, and levels of PaO2 decreased significantly when rats were treated with common bile duct ligation (CBDL). We also selected the putative microRNA binding site on PAI-1 by bioinformatics analysis. Our data indicate that miR145-5p is a microRNA binding site on PAI-1. Then, miR145-3p and miR145-5p expression levels in the lungs and PMVECs of rats were detected by qRT-PCR because miR145-3p and miR145-5p are produced by a precursor, miR145. Our findings revealed that miR145-3p is very weakly expressed in lung, and treatment with CBDL or rat serum did not change expression levels of miR145-3p in lungs or PMVECs of rats. However, expression levels of miR145-5p in lungs decreased significantly in a time-dependent manner in rats after CBDL treatment, and the same change occurred in expression levels of miR145-5p in PMVECs when cells were exposed to CBDL rat serum. In addition, we confirmed that miR145-5p is a negative regulator or PAI-1 expression using a luciferase reporter system. Furthermore, we used Ki67 staining and Cell Counting Kit-8 (CCK-8) assay to analyze miR145-5p regulation of PMVEC proliferation. Our results showed that a miR145-5p mimic significantly inhibited PMVEC proliferation. Our findings indicate that miR145-5p inhibits proliferation of PMVECs via PAI-1 in experimental hepatopulmonary syndrome rat pulmonary microvascular hyperplasia. We believe that PAI-1 and miR145-5p play key roles in HPS-associated IPVD and could represent effective therapeutic targets.

## RESULTS

### Significant pulmonary microvascular hyperplasia was found in lung tissue of HPS rats

**Fig. 1.**
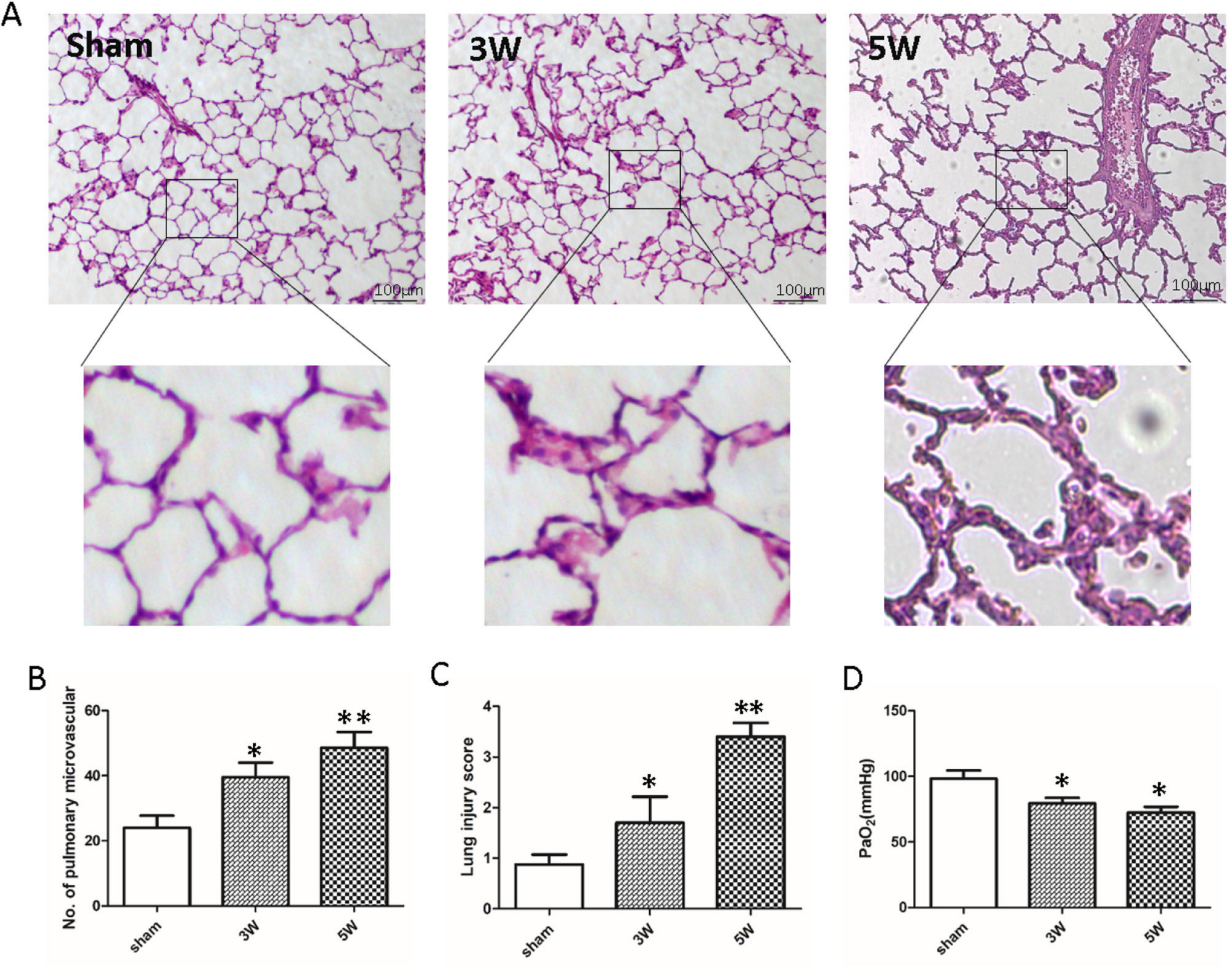
**CBDL** **surgery induces pulmonary injury.** (A) Histology assessment of lungs in rats after CBDL surgery or sham procedure (*n*=15; scale bars: 100 µm). Magnifications show pulmonary microvascular. (B) Number of pulmonary microvascular in the lungs of CBDL rats or rats from the sham group (*n*=15). (C) Lung injury score of the lungs from rats after CBDL surgery or sham procedure (*n*=15). (D) PaO_2_ gradually decreased after CBDL surgery in rats (*n*=15) (compared with sham group; *P<0.05, **P<0.01).

First, an HPS rat model was constructed using CBDL. After surgery, lung alveolar epithelial cells were flattened, and alveoli became more unstable compared to the rats in the sham group when observed with Hematoxylin and Eosin (H&E) staining. We counted the number of pulmonary microvascular in the different fields of view from all groups in the same area. Results showed that the number of pulmonary microvascular in the lungs of CBDL rats increased significantly compared to the lungs of sham control rats (Fig. 1A,B). H&E staining results revealed that the lung injury score of CBDL rats increased gradually with time (Fig. 1C). Compared to sham rats, the level of PaO_2_ in CBDL rats was significantly decreased (Fig. 1D). These results show that arterial oxygenation was impaired in the setting of pulmonary microvascular hyperplasia.

### mRNA and protein expression levels of PAI-1 are upregulated in the HPS rat lung

**Fig. 2.**
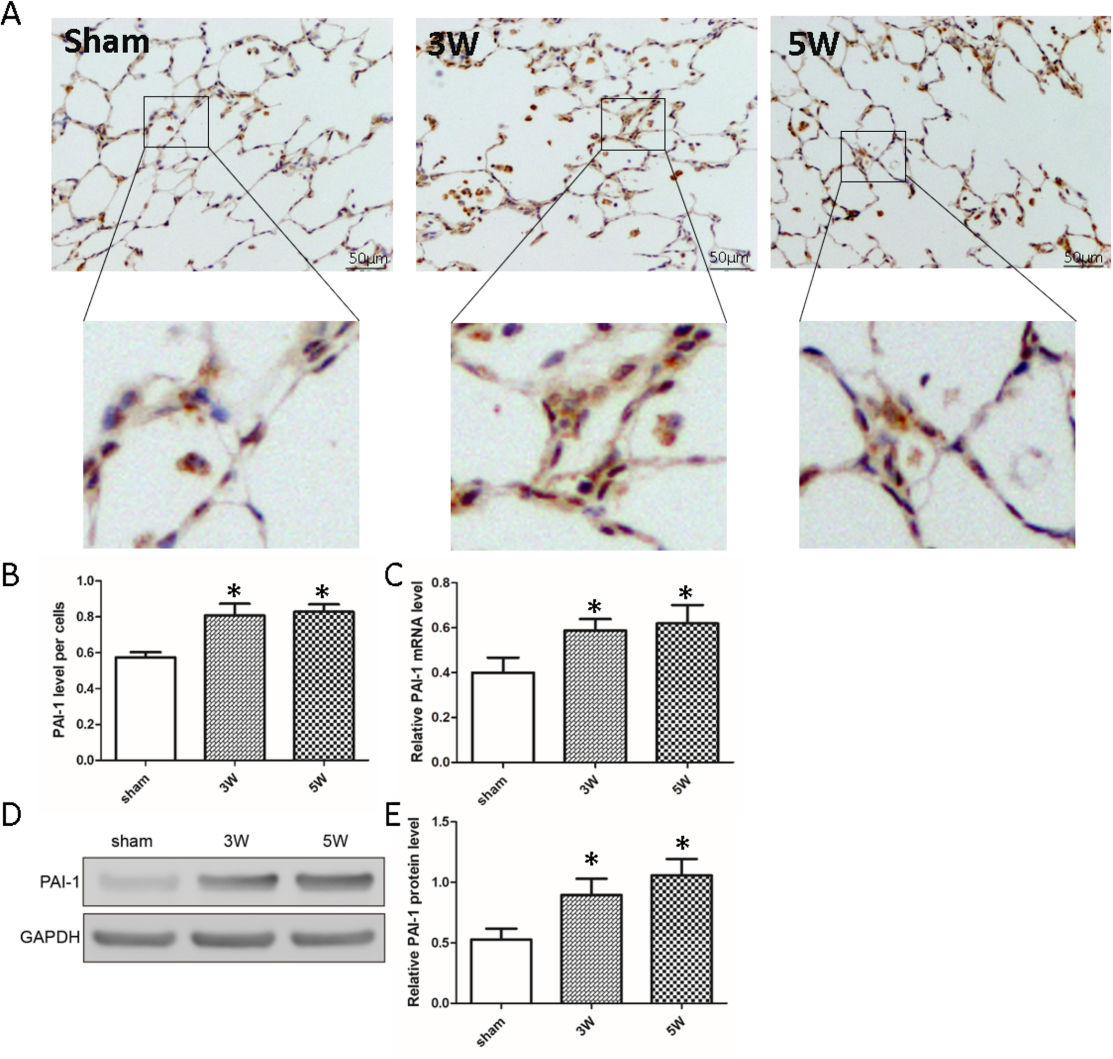
**PAI-1 expression is induced in CBDL rat lungs during progression of HPS.** (A) Immunohistochemistry detected PAI-1 synthesis in the lungs of the rats after CBDL surgery (3 or 5 weeks) or sham procedure. Scale bars: 50 µm. Magnifications show PAI-1 expression in pulmonary microvascular. (B) Statistics of PAI-1 relative expression level per PMVEC of different rat lungs (*n*=15). (C) qRT-PCR detection of PAI-1 gene transcription levels in the lungs of different groups (*n*=15). (D,E) Western blot detection of PAI-1 synthesis in the lungs of rats after CBDL surgery (3 or 5 weeks) or sham procedure (*n*=6) (compared with sham group; *P<0.05).

Immunochemistry analysis demonstrated that PAI-1 was weakly expressed in the lungs of rats that underwent a sham procedure (sham group). However, expression of PAI-1 increased (*P*<0.05) in the lungs of rats that underwent CBDL as time increased (CBDL3w, CBDL5w) (Fig. 2A,B). qRT-PCR results showed that PAI-1 mRNA levels remained low in the lungs of sham control rats. However, following treatment with CBDL, mRNA levels of PAI-1 in the lungs gradually increased over time (Fig. 2C). Western blotting assay demonstrated that expression levels of PAI-1 in the lungs of CBDL rats increased significantly compared to the lungs of sham control rats (Fig. 2D,E). These results indicate that PAI-1 synthesis is increased in experimental HPS rat pathological IPVD.

### MiR145-5p has a microRNA binding site on PAI-1, and CBDL treatment induces changes in the expression levels of miR145-3p and miR145-5p in the lungs and PMVECs of rats

We predicted the putative microRNA binding site on PAI-1 using two microRNA online websites. The putative microRNA binding site on the PAI-1 gene was predicted by bioinformatics analysis. From these results, we found that miR145-5p has a microRNA binding site on PAI-1 (Table S3). We hypothesized that miR145-5p plays a key role in HPS-associated IPVD by regulating PAI-1 expression, for miR-145-3p and miR-145-5p inhibit cell proliferation and migration in multiple diseases, including lung and gastric cancer. Furthermore, miR145-3p and miR145-5p are produced by a precursor, miR145.

**Fig. 3.**
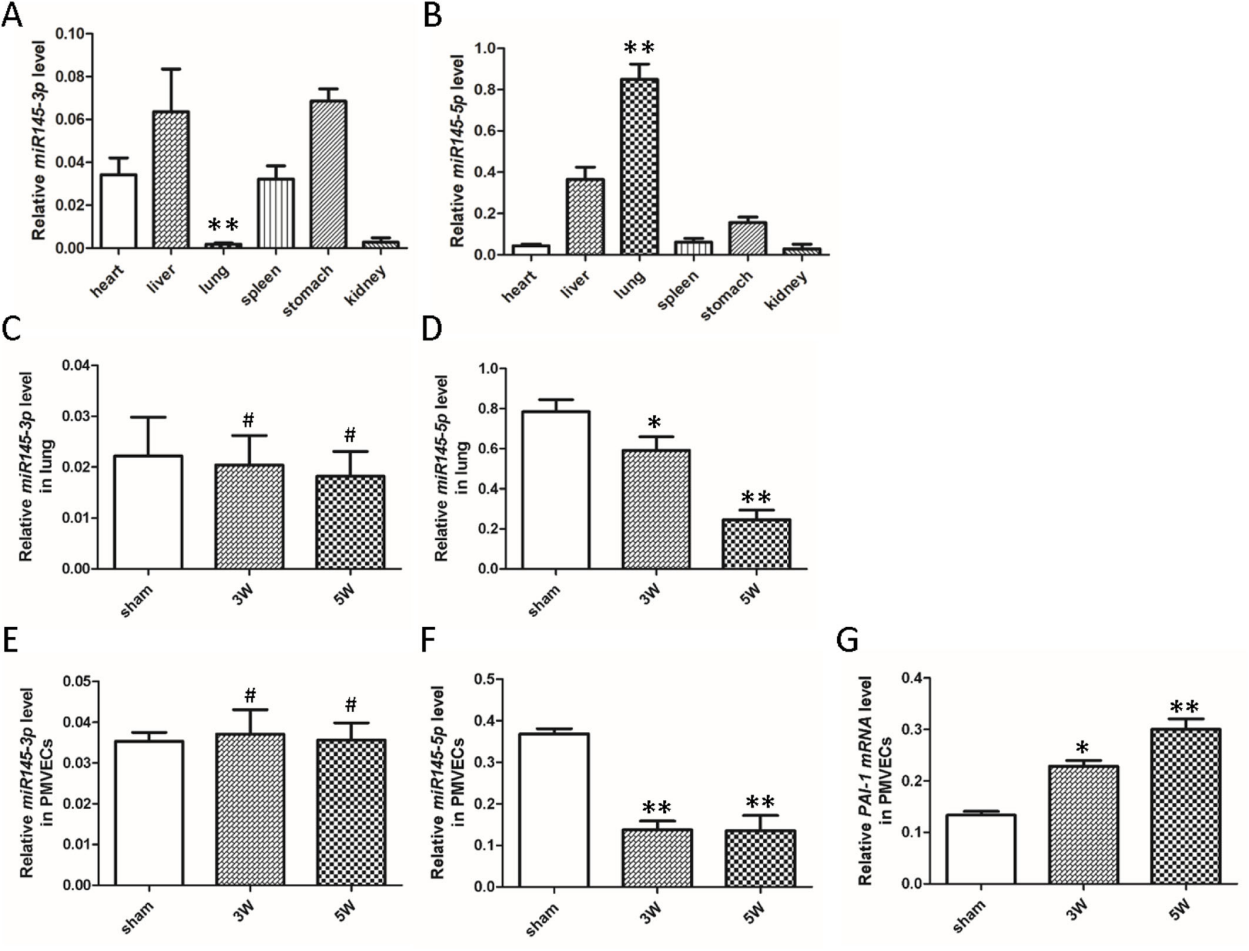
**Changes in miR145-3p and miR145-5p levels in normal rat internal organs, CBDL rat lungs and CBDL rat serum-treated PMVECs.** (A) MiR145-3p was only weakly expressed in lungs compared to other organs (*n*=6). (B) Expression levels of miR145-5p were the highest in lung tissue and more than twice those in the liver (*n*=6). (C) qRT-PCR detected expression levels of miR145-3p in the lungs of rats after CBDL surgery (3 or 5 weeks) or sham procedure (*n*=6). (D) Expression levels of miR145-5p in lungs significantly decreased in a time-dependent manner in rats after CBDL (*n*=6). (E) qRT-PCR detection of expression levels of miR145-3p in PMVECs treated with different serum (*n*=6). (F) Expression of miR145-5p in PMVECs decreased significantly after cells were exposed to CBDL rat serum (*n*=6). (G) After exposure to CBDL rat serum, transcription levels of PAI-1 in PMVECs increased in a time-dependent manner (*n*=6) (A,B: compared with other groups, **P<0.01; C–G: compared with sham group, ^#^*P*>0.05, *P<0.05, **P<0.01).

Next, we detected expression levels of miR145-3p and miR145-5p in the lungs and PMVECs of rats in all groups. qRT-PCR results revealed that miR145-3p was only weakly expressed in lungs compared to other organs (Fig. 3A). However, expression levels of miR145-5p were the highest in lung tissue, more than twice those seen in the liver (Fig. 3B). Results showed that treatment with sham procedure or CBDL did not change the expression level of miR145-3p in lungs, which remained consistently low in each group (Fig. 3C). However, expression levels of miR145-5p in lungs significantly decreased in a time-dependent manner when in rats after CBDL (Fig. 3D). qRT-PCR results showed that expression levels of miR145-3p in PMVECs treated with different serum remained consistently low no matter the serum stimulation (Fig. 3E). Results also showed that treatment with serum in sham rats did not decrease miR145-5p expression in PMVECs, but after exposure to CBDL rat serum, expression of miR145-5p decreased significantly in PMVECs (Fig. 3F). Meanwhile, we found that after exposure to CBDL rat serum, transcription levels of PAI-1 in PMVECs increased in a time-dependent manner (Fig. 3G).

### MiR145-5p inhibits expression of PAI-1

**Fig. 4.**
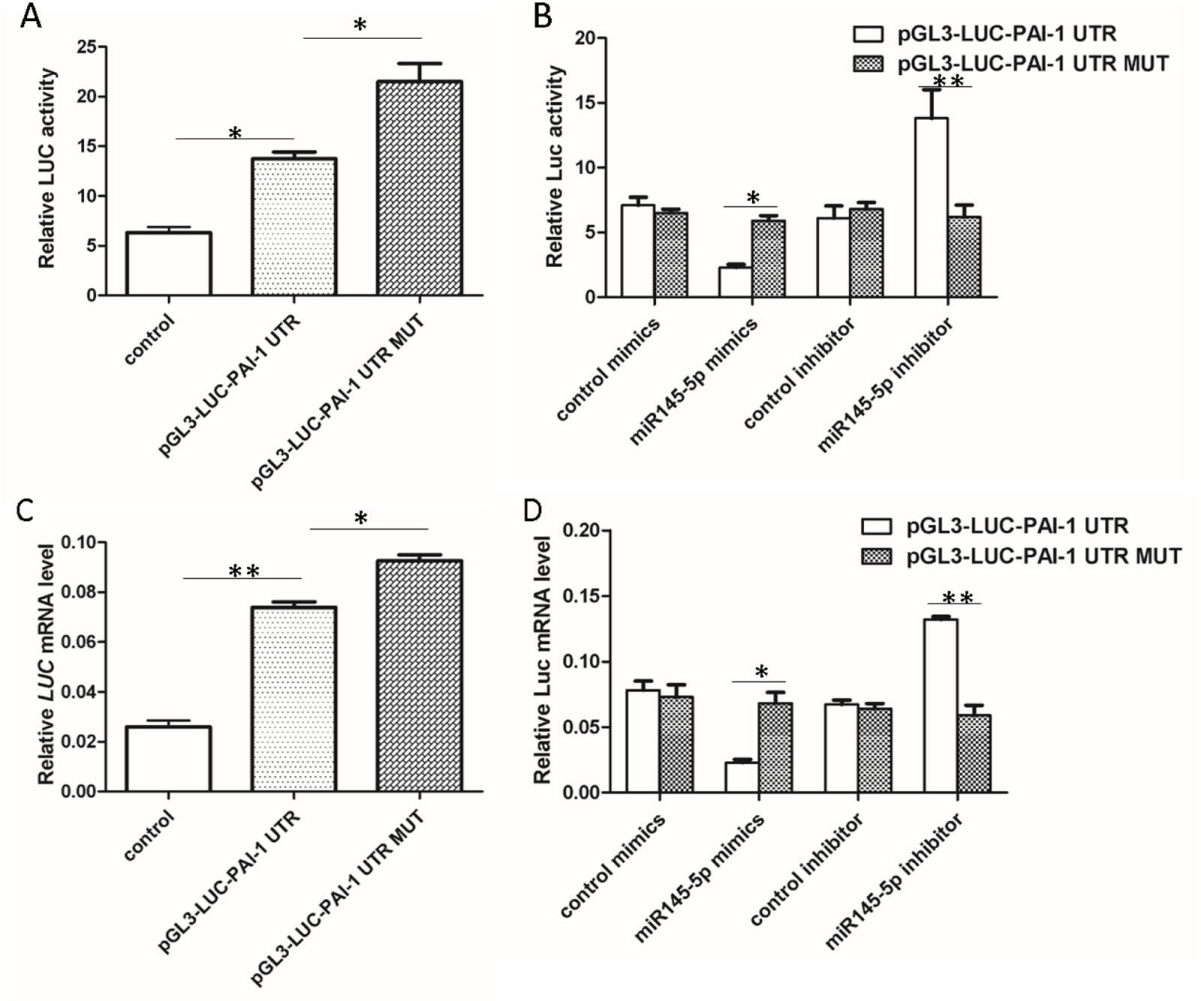
**Identification of PAI-1 as a direct target of miR145-5p in PMVECs.** (A,B) Luciferase activity was analyzed 48 h after PMVECs were co-transfected with pGL3-UC-PAI-1 UTR (or pGL3-UC-PAI-1 UTR MUT) and miR145-5p mimics, control mimics, miR145-5p inhibitor or control inhibitor (*n*=3). (C,D) qRT-PCR analysis of Luc gene transcription in PMVECs after co-transfection with pGL3-UC-PAI-1 UTR (or pGL3-UC-PAI-1 UTR MUT) and miR145-5p mimics, control mimics, miR145-5p inhibitor or control inhibitor (*n*=3) (A,C: compared with pGL3-UC-PAI-1 UTR or control, *P<0.05, **P<0.01; B,D: compared with pGL3-UC-PAI-1 UTR MUT, *P<0.05, **P<0.01).

To analyze the function of the miR145-5p-targeted sequence in the 3′ UTR of PAI-1, we constructed a luciferase reporter system as previously descried. Results demonstrated that there was higher Luc activity in PMVECs transfected with pGL3-LUC-PAI-1 UTR and pGL3-LUC-PAI-1 UTR MUT (Fig. 4A). Then, PMVECs were treated with miR145-5p mimic and inhibitor. qRT-PCR results revealed that the miR145-5p mimic reduced Luc activity by two thirds, and the miR145-5p inhibitor enhanced Luc activity twofold (Fig. 4B).

**Fig. 5.**
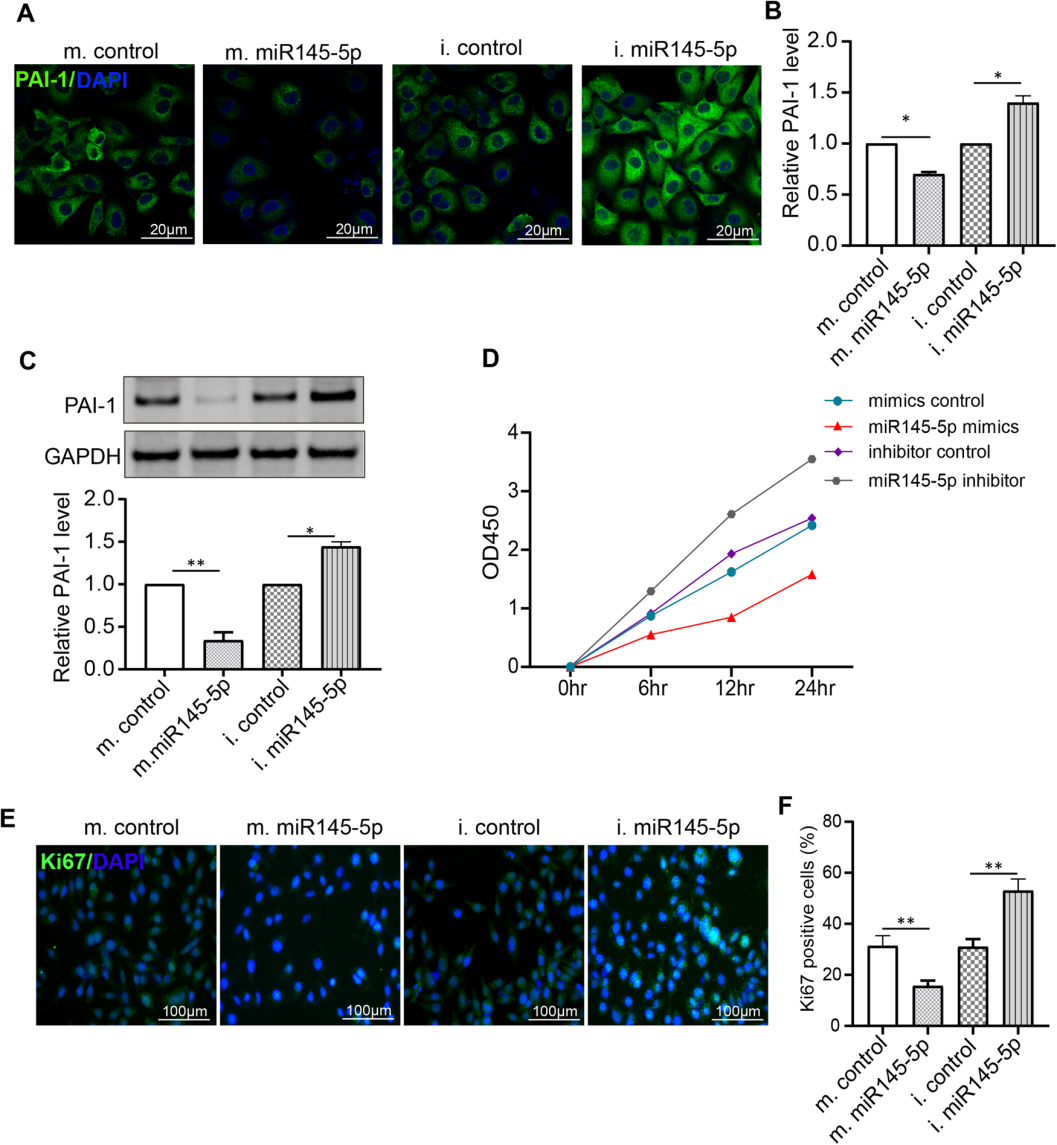
**Identification of miR145-5p as a negative regulator of PAI-1.** (A,B) Immunofluorescence staining analyzed PAI-1 expression after PMVECs were transfected with miR145-5p mimics or inhibitor. Scale bars: 20 µm. (C) Western blot analyzed PAI-1 expression after PMVECs were transfected with miR145-5p mimics or inhibitor. Overexpression of miR145-5p inhibits PMVEC proliferation. (D) OD450 values detected by CCK-8 assay statistics. *P*<0.05, mimic control compared with miR144-5p mimics; inhibitor control compared to the miR145-5p inhibitor. (E) Immunofluorescence staining of Ki67 showing positive proliferation of PMVECs after treatment with miR144-5p mimics, mimic controls, miR145-5p inhibitor or inhibitor control. Scale bars: 100 µm. (F) Ki67-positive cell statistics. m, mimic; i, inhibitor. *n*=3, *P<0.05, **P<0.01, compared to miR144-5p mimic or miR145-5p inhibitor.

Furthermore, we detected mRNA levels of Luc in PMVECs when cells were transfected with different vectors and miRNA. Results showed that transcription levels of Luc in PMVECs significantly increased when cells were transfected with pGL3-LUC-PAI-1 UTR and pGL3-LUC-PAI-1 UTR MUT (Fig. 4C). In addition, qRT-PCR results demonstrated that the miR145-5p mimic reduced transcription levels of Luc by two thirds and the miR145-5p inhibitor induced transcription levels of Luc twofold (Fig. 4D). The immunofluorescence results showed that after miR145-5p mimic transfection, the expression of PAI-1 decreased significantly in PMVECs compared with cells treated with mimic control. The expression of PAI-1 increased dramatically in PMVECs following treatment with miR145-5p inhibitor compared with cells transfected with inhibitor control (Fig. 5A,B); the western blot results confirmed it (Fig. 5C). These results indicate that miR145-5p is a negative regulator of PAI-1 expression by targeting PAI-1 3′ UTR.

### MiR145-5p inhibits PMVEC proliferation by downregulating the expression of PAI-1

We used CCK-8 assay and Ki67 staining to analyze miR145-5p regulation of PMVEC proliferation. The results of the CCK-8 assay demonstrated that miR145-5p mimic significantly inhibits PMVEC proliferation. After exposure of PMVECs to a miR145-5p mimic, there was significant inhibition of PMVEC proliferation in the miR145-5p mimic compared to the other groups. In contrast, the miR145-5p inhibitor had the opposite effect on PMVEC proliferation compared to the miR145-5p mimic (Fig. 5D). The results of Ki67 staining showed that when miR145-5p was overexpressed by mimic, PMVEC proliferation was reduced (Fig. 5E,F). When miR145-5p was reduced by an miR145-5p inhibitor, PMVEC proliferation was improved (Fig. 5E,F).

**Fig. 6.**
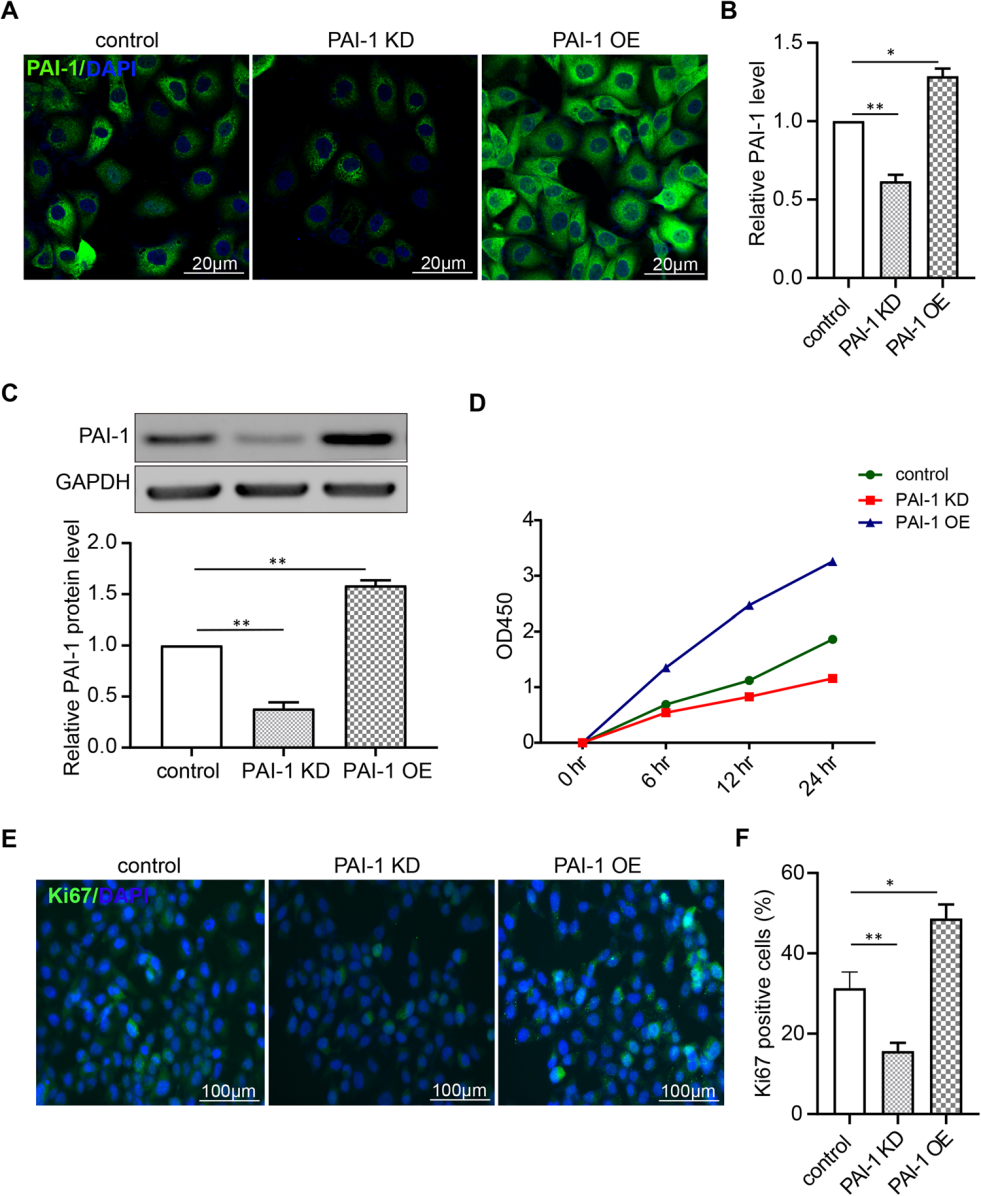
**PAI-1 promotes PMVECs proliferation.** (A,B) Immunofluorescence staining was analyzed 48 h after PMVECs were transfected with PAI-KD, PAI-OE or control. Scale bars: 20 µm. (C) Western blot was analyzed 48 h after PMVECs were transfected with PAI-KD, PAI-OE or control. Downregulating of PAI-1 expression inhibits PMVEC proliferation. (D) OD450 values detected by CCK-8 assay statistics. *P*<0.05, PAI-KD group compared with control group; PAI-1-OE group compared to control group. (E) Immunofluorescence staining of Ki67 showing positive proliferation of PMVECs after treatment with PAI-KD, PAI-1 OE or PAI-1 control. Scale bars: 100 µm. (F) Ki67-positive cell statistics. (*n*=3, *P<0.05, **P<0.01, compared to PAI-KD group).

Further, we used PAI-1 specific lentivirus transfection to alter the expression of PAI-1 to mimic the effects of miR145-5p on PAI-1. The immunofluorescence results showed that when transfected with PAI-1 KD, the expression of PAI-1 in PMVECs decreased significantly compared with cells treated with lentivirus control. In contrast, the PAI-1 OE had the opposite effect on the expression of PAI-1 compared to the PAI-1 KD (Fig. 6A,B). The western blot results showed that the expression of PAI-1 decreased dramatically in PAI-1 KD group and increased significantly in PAI-1 OE group compared with the control group (Fig. 6C). The results of CCK-8 assay and Ki67 staining showed that when the PMVECs transfected with PAI-1-KD, cell proliferation was reduced by downregulating the expression of PAI-1. When the PMVECs were transfected with PAI-1-OE, cell proliferation was improved by upregulating the expression of PAI-1 (Fig. 6D–F). These results indicate that miR145-5p affects PMVEC proliferation by regulating the expression of PAI-1.

## DISCUSSION

Clinically, HPS presents as abnormal dilation of pulmonary micro vessels, as well as abnormalities in both gas exchange and arterial oxygenation based on chronic liver disease and/or portal hypertension (Swanson et al., 2005). It has been reported that patients with HPS experience a twofold risk of mortality compared to cirrhotic patients without HPS, independent of the severity of cirrhosis. Gradually progressive dyspnea (annual decline in PaO_2_ of 5 mmHg/year) is regarded as the most representative symptom of patients with HPS (Fallon et al., 2008). The major pathology of HPS includes IPVD, which leads to development of hypoxemia. At present, the pathophysiological mechanism underlying HPS-related IPVD is unclear, and liver transplantation is currently the only treatment option (Grace and Angus, 2013).

Our data demonstrate that the number of pulmonary microvascular in lung tissue of HPS rats increased significantly, while the lung injury score was noticeably upregulated, with levels of PaO_2_ significantly decreased in rats treated with CBDL. Immunochemistry and western blot analyses indicate that expression of PAI-1 in lung tissue of HPS rats was increased significantly. PAI-1 is primarily produced by the endothelium but is also secreted by other tissue types such as adipose tissue. Congenital deficiency of PAI-1 has been reported, and as fibrinolysis is not suppressed adequately, it leads to hemorrhagic diathesis. PAI-1 is present in increased levels in various disease states, such as multiple forms of cancer, as well as in obesity and metabolic syndrome (Pusina, 2018). It has been linked to increased occurrence of thrombosis in patients with these conditions. Previous studies have demonstrated that some factors, such as high glucose, induce vascular smooth muscle cell (VSMC) proliferation accompanied by upregulation of PAI-1 expression (Jeong et al., 2011; Yuan and Liu, 2011). Our findings confirm the occurrence of pulmonary microvascular hyperplasia in HPS-associated IPVD. Meanwhile, due to the role of PAI-1 in regulating PASMC phenotypic modulation and pulmonary vascular permeability, we hypothesized reasonably that it plays an important role in HPS-associated pulmonary microvascular hyperplasia.

It is widely accepted that miRNAs regulate protein translation through their target messenger RNAs (mRNAs) via sequence-specific interaction to repress translation or degrade target mRNAs (de Lucia et al., 2017). Some miRNAs have been found circulating in body fluids, but most miRNAs are located within cells. Thus, miRNA-mediated regulation is an important post-transcriptional gene regulation mechanism and is estimated to modulate up to 30% of mammal genes and has an important role in human cardiovascular function (Perez-Cremades et al., 2018). It is reported that some miRNAs, such as miRNA-101, inhibit proliferation of PMVECs in an HPS rat model (Wang et al., 2015). The adjustment function of PAI-1 on changes in pathology of HPS-associated IPVD may be related to miRNAs. Hence, we identified a putative microRNA binding site on PAI-1 by bioinformatics analysis. Our data show that miR145-5p has a microRNA binding site on PAI-1.

It is reported that miR145-3p and miR145-5p are produced by a precursor, miR145, and they play similar regulatory roles in the pathological process of many diseases. A number of studies have demonstrated that miR145-3p apparently controls cell proliferation, whereas the activity of miR145-5p seems to modulate cell migration and invasion. Downregulation of miR145-3p and 145-5p is related to the risk of breast cancer, but their roles in HPS are not yet clarified (Muti et al., 2014). Although research on the true biologic relevance of miR145-5p in HPS is still in its infancy, in this study, we focused on the expression and function of miR145-5p in the angiogenesis of HPS. Then, we detected miR145-3p and miR145-5p expression levels in the lungs and PMVECs of rats by qPT-PCR. Our findings show that miR145-3p was weakly expressed in lungs of rat, and treatment with CBDL procedure or rat serum did not change expression levels of miR145-3p in lungs or PMVECs of rats. However, expression levels of miR145-5p in lungs were significantly decreased in rats after CBDL. The same change occurred in expression levels of miR145-5p in PMVECs treated with CBDL rat serum. We hypothesized reasonably that miR145-5p may regulate the PAI-1 signaling pathway to control pathogenesis of HPS-associated IPVD.

In addition, we confirmed that miR145-5p is a negative regulator of PAI-1 expression using western blotting, immunofluorescence and a luciferase reporter system (Yang et al., 2018). Due to the most important pathological change of HPS-associated pulmonary microvascular hyperplasia in the *in vitro* experimental HPS model being abnormal proliferation of PMVECs, we used Ki67 staining and CCK-8 assay to analyze miR145-5p regulation of PMVECs proliferation. Results revealed that the miR145-5p mimic significantly inhibited PMVEC proliferation.

In conclusion, our data confirm the occurrence of pulmonary microvascular hyperplasia in HPS. Expression of PAI-1 in lungs increased significantly, while miR145-5p expression in lungs was noticeably suppressed in rats after CBDL. The same change in expression levels of miR145-5p occurred in PMVECs exposed to CBDL rat serum. Upon transfection with miR145-5p mimic, we observed reduced PMVEC proliferation. And the regulation of PMVEC proliferation by miR-145-5p is achieved by PAI-1, for the expression changes of PAI-1 directly affect PMVEC proliferation. This study shows the direct involvement of miR145-5p, PAI-1 and PMVECs in HPS rat pulmonary microvascular hyperplasia. Our findings provide a proof of principle that microRNAs may be useful for the future development of novel therapeutic strategies in HPS. Nonetheless, further investigations using miR145-5p knockout animal models must be done to validate our findings. In the future, it will be useful to understand the mechanisms of abnormal cell proliferation to provide a basis for inhibiting pulmonary microvascular hyperplasia and creating targeted therapies for diseases associated with IPVD.

## MATERIALS AND METHODS

### Animals

This study was performed in accordance with guidelines from the National Institutes of Health. All procedures performed on rats were approved by the Animal Care Committee of Third Military Medical University. Sprague Dawley (SD) rats weighing 180–220 g, which were obtained from the laboratory animal center of the Third Military Medical University, were used for experiments.

### HPS rat model

The HPS rat model was induced by CBDL as a well-established methodology in our initial study (Liu et al., 2017). Forty-five rats were selected to undergo surgery. The experimental group (*n*=30) underwent CBDL. The control group (*n*=15) underwent a sham procedure. Lungs of the animals were dissected and analyzed 3 and 5 weeks after surgery. A blood sample was aseptically drawn from the abdominal aorta during laparotomy. A 0.2 ml arterial blood sample was used to measure arterial gas levels. Serum was separated from the blood samples of rats in the two groups at the same time. All serum samples were used for subsequent experiments. The edges of the lung tissues from the control and experimental rats were collected for histopathological examination and western blotting.

### Cell culture

PMVECs were isolated from the lungs of SD rats as previously described (Yang et al., 2018). Briefly, lung tissue was rinsed in 10 mM phosphate-buffered saline (PBS), finely minced into small pieces and digested in 0.3% type II collagenase (Thermo Fisher Scientific) for 45 min with occasional agitation. The cell pellet was resuspended in binding buffer and incubated for 20 min at 4°C with magnetic microbeads (Invitrogen) coated with anti-CD31 antibody. Isolated cells were resuspended in 10% Endothelial Growth Medium-2 (EGM-2, Sigma-Aldrich) and incubated for 8–12 h at 37°C. 7–10 days after seeding the cells, fast growing fibroblasts in the flask were repeatedly removed mechanically using the sharp end of a rubber rod under direct microscopy. When PMVECs proliferated to 50% confluence, they were harvested with trypsin-EDTA and repurified using anti-CD31-coated magnetic microbeads as previously described. PMVECs were passaged at a ratio of 1:3 and used for experiments at passages 2–3. PMVECs of the same concentration (10^6^/cm^2^) were inoculated in flask bottoms. When cells covered 80% of the flask bottom, the original serum was replaced with 0.1% fetal bovine serum (FBS; Gibco, San Diego, USA). After 24 h of synchronous growth, PMVECs were treated for 24 h with serum (5%) obtained from rats after CBDL surgery (3 or 5 weeks) or sham procedure. After treatment, cells were used for subsequent experiments.

### Bioinformatics analysis

Potential targets of microRNA145-3p and microRNA145-5p were predicted using Target Scan Human (http://www.targetscan.org/) and miRTarBase (http://mirtarbase.mbc.nctu.edu.tw).

### qRT-PCR

Total RNAs were extracted from lung tissues or PMVECs using the miRNeasy Mini Kit (Qiagen, Hilden, Germany) and quantified using a NanoDrop spectrophotometer (Thermo Fisher Scientific). Template RNA was reverse-transcribed to cDNA using the M-MLV reverse transcriptase (Promega, Madison, USA). MiRNA specific stemloop primers, oligo(dT), or random primers were added to initiate cDNA synthesis. A SYBR^®^ Green Quantitative RT-qPCR Kit (Sigma-Aldrich) was used to perform qRT-PCR. Relative levels of mRNA of target genes were calculated with the 2−ΔΔCt method, and glyceraldehyde-3-phosphate dehydrogenase gene Ct values were calculated according to the manufacturer's instructions. Experiments were performed at least three times. Primers for qPCR are shown in Table S1.

### Western blot

Lung tissues were dissected and lysed in radio-immunoprecipitation assay lysis buffer (Beyotime, Shanghai, China). BCA protein assay (CWBIO, Beijing, China) was used to quantify the amount of protein. Equal quantities of protein were analyzed using sodium dodecyl sulfate-polyacrylamide gel electrophoresis (SDS-PAGE, CWBIO, Beijing, China) and transferred to PVDF membranes. The membranes were blocked for 1 h using a blocking solution. Samples were incubated overnight with anti-PAI-1 (1:1000, no. ab66705, Abcam) and anti-GAPDH (1:2500, no. AF1186, Beyotime, Shanghai, China). The membrane was washed twice with TBS, and bound antibody was detected using IgG-HRP secondary antibodies, HRP-conjugated goat anti-rabbit IgG and HRP-conjugated goat anti-mouse IgG (no. A0208 and no. A0216, Beyotime, Shanghai, China), at a ratio of 1:1000 for 2 h at room temperature. Finally, membranes were stained with ECL reagent (Beyotime, Shanghai, China), scanned, and stored using a gel imaging system. Optical density of the immunoreactivity was measured and analyzed with an Alpha Imager.

### Vector construction

The 3′UTR region of the rat PAI-1 gene (NM_012620) was amplified from rat lung cDNA, the primer with Xba I and BamH I restriction enzyme sites, and cloned into pMD-19T simple vectors (TaKaRa Biotech, Kusatsu, Japan) for sequencing. Fragments were digested by Xba I and BamH I and transferred to a luciferase reporter plasmid pGL3 promoter vector (Promega). The reporter vector was named pGL3-LUC-PAI-1 UTR. The mutant vectors with the miR145-5p binding site in the PAI-1 UTR mutant were constructed by site-specific mutagenesis based on the pGL3-LUC-PAI-1 UTR vector. The entire vector was amplified by a special primer, which contains the nucleic acids mutated in the target site, and the template vector and the mutation vector were transformed into *Escherichia coli* competent cells. Then, sequencing and plasmid preparation for cell transfection were completed. All primers are listed in Table S1.

### Transfection and dual-luciferase reporter assay

The transfection assay was performed with the Dual-Luciferase Reporter Assay System (Promega) in the GloMax-Multi Detection system Photometer (Promega). MiR145-5p mimic, miR145-5p inhibitor, control mimic and inhibitor were synthesized by Gene RIB Bio (Guangzhou, China). 24 h before transfection, cells were seeded into 24-well plates at 1×10^5^ cells/well. MiR145-5p mimic (50 nM) or miR145-5p inhibitor (100 nM) were transfected into the PMVECs using Lipofectamine 3000 (Invitrogen). For luciferase assays, each wild-type or deletion vector was transfected into the cells at 500 ng, together with 50 ng/well of pRL-TK (Promega). 24 h after transfection, luciferase activities were measured with a VARIOSKAN FLASH (Thermo Fisher Scientific). The experiment was performed three times independently, and the average expression levels of the target genes are presented as the mean±s.e.m.

### Immunochemistry

5 μm sections of 10% formalin paraffin-fixed lung tissues were deparaffinized, retrieved, blocked with 5% serum and incubated with anti-PAI-1 (1:1000, no. ab66705, Abcam) followed by horseradish peroxidase conjugated secondary antibodies. Positive signals were detected using a Diaminobenzidine Peroxidase Substrate Kit (DAKO, Glostrup, Denmark) and then counterstained with H&E. The microphotographs of sections were taken with a light microscope (Olympus) and quantified by Image-Pro plus 6.010 (Media Cybernetics, Silver Spring, USA) followed by immunochemistry data analysis protocol.

### Immunofluorescence

5 μm-thick paraffin sections of fixed PMVECs were blocked in 10% bovine serum albumin for 2 h. Next, sections were incubated overnight at 4°C with anti-Ki67 (1:1000, no. ab15580, Abcam) and anti-PAI-1 (1:1000, no. ab31280, Abcam). Then, cells were incubated in a 1:500 dilution of fluorescence-tagged secondary antibody for 2 h. After washing twice with PBS, PMVECs were incubated with DAPI for 10 min. Cells were then washed with PBS for 5 min and mounted on coverslips using a drop of the anti-fade mounting medium. Sections were investigated using a confocal microscope.

### Histological analysis

Sections were stained with H&E. The morphology score and number of pulmonary microvascular were assessed according to Cui et al. (2015) and Yang et al. (2018).

### CCK-8 assay

Cell proliferation was detected by the CCK-8 assay according to the manufacturer's instructions. At the end of serum treatment, cells in 96-well plates (100 μl of medium) were incubated with 10 μl CCK-8 solution (Dojindo Laboratories, Kumamoto, Japan) for 2 h at 37°C. Next, viable cells were detected by measuring the absorbance values at 450 nm using the Varioskan Flash Multimode Reader (Thermo Fisher Scientific).

### PAI-1 knockdown and overexpressed lentiviral transduction

PAI-1 knockdown lentivirus and overexpressed lentivirus were made by GenePharma Inc (Shanghai, China). A lentivirus empty vector was used as a control. The sequence information for siRNA targets PAI-1 was provided in Table S2. Inoculated PASMCs of the same concentration (10^6^/cm^2^) were divided into a PAI-1 shRNA-transfected group (PAI-1 knockdown), lentivirus PAI-1-transfected group (PAI-1 overexpressed) and a lentivirus empty vector group (control). When the cells covered 80% of the flask bottom, the original serum was replaced with 0.1% FBS for 24 h of synchronous growth. PAI-1 knockdown, PAI-1 overexpressed and control, at a multiplicity of infection (MOI) of 20, were added into the medium of the corresponding groups. The flask of these groups were placed upside-down on an inverted immunofluorescence microscope to observe the transfection efficiency. And the efficacy of lentivirus on the different groups of cells was assessed using western blotting after 24 h of transfection.

### Statistical analysis

Statistical analysis was performed with SPSS23.0 (IBM, USA). The two-tailed *t*-test was applied for comparison between two groups. Data are shown as the mean±standard error. One-way analysis of variance was adopted for comparison of multiple samples. A value of *P*<0.05 was considered statistically significant.

## Supplementary Material

Supplementary information

## References

[BIO044800C1] Bartel D. P. (2009). MicroRNAs: target recognition and regulatory functions. *Cell* 136, 215-233. 10.1016/j.cell.2009.01.002 19167326 PMC3794896

[BIO044800C2] Chen L., Li Y.-S., Cui J., Ning J.-N., Wang G.-S., Qian G.-S., Lu K.-Z. and Yi B. (2014). MiR-206 controls the phenotypic modulation of pulmonary arterial smooth muscle cells induced by serum from rats with hepatopulmonary syndrome by regulating the target gene, annexin A2. *Cell. Physiol. Biochem.* 34, 1768-1779. 10.1159/000366377 25427750

[BIO044800C3] Chen T., Huang J. B., Dai J., Zhou Q., Raj J. U. and Zhou G. (2018). PAI-1 is a novel component of the miR-17∼92 signaling that regulates pulmonary artery smooth muscle cell phenotypes. *Am. J. Physiol. Lung Cell. Mol. Physiol.* 315, L149-L161. 10.1152/ajplung.00137.2017 29644896 PMC6139661

[BIO044800C4] Cui J., Zhao H., Yi B., Zeng J., Lu K. and Ma D. (2015). Dexmedetomidine attenuates bilirubin-induced lung alveolar epithelial cell death in vitro and in vivo. *Crit. Care Med.* 43, e356-e368. 10.1097/CCM.0000000000001035 26274721 PMC4535733

[BIO044800C5] de Faria C. A., Zanette D. L., Silva W. A. Jr and Ribeiro-Paes J. T. (2019). PAI-1 inhibition by simvastatin as a positive adjuvant in cell therapy. *Mol. Biol. Rep.* 46, 1511-1517. 10.1007/s11033-018-4562-4 30612281

[BIO044800C6] de Lucia C., Komici K., Borghetti G., Femminella G. D., Bencivenga L., Cannavo A., Corbi G., Ferrara N., Houser S. R., Koch W. J. et al. (2017). microRNA in cardiovascular aging and age-related cardiovascular diseases. *Front. Med. (Lausanne)* 4, 74 10.3389/fmed.2017.00074 28660188 PMC5466994

[BIO044800C7] Fallon M. B., Krowka M. J., Brown R. S., Trotter J. F., Zacks S., Roberts K. E., Shah V. H., Kaplowitz N., Forman L., Wille K. and et al. (2008). Impact of hepatopulmonary syndrome on quality of life and survival in liver transplant candidates. *Gastroenterology* 135, 1168-1175. 10.1053/j.gastro.2008.06.038 18644373 PMC2824882

[BIO044800C8] Filipowicz W., Bhattacharyya S. N. and Sonenberg N. (2008). Mechanisms of post-transcriptional regulation by microRNAs: are the answers in sight? *Nat. Rev. Genet.* 9, 102-114. 10.1038/nrg2290 18197166

[BIO044800C9] Gao J., Chen L., Zeng J., Cui J., Ning J.-L., Wang G.-S., Belguise K., Wang X., Qian G.-S., Lu K.-Z. and et al. (2015). The involvement of aquaporin 1 in the hepatopulmonary syndrome rat serum-induced migration of pulmonary arterial smooth muscle cells via the p38-MAPK pathway. *Mol. Biosyst.* 11, 3040-3047. 10.1039/C5MB00347D 26315345

[BIO044800C10] Grace J. A. and Angus P. W. (2013). Hepatopulmonary syndrome: update on recent advances in pathophysiology, investigation, and treatment. *J. Gastroenterol. Hepatol.* 28, 213-219. 10.1111/jgh.12061 23190201

[BIO044800C11] He J., Yi B., Chen Y., Huang Q., Wang H., Lu K. and Fu W. (2017). The ET-1-mediated carbonylation and degradation of ANXA1 induce inflammatory phenotype and proliferation of pulmonary artery smooth muscle cells in HPS. *PLoS ONE* 12, e0175443 10.1371/journal.pone.0175443 28414743 PMC5393570

[BIO044800C12] Ivey K. N. and Srivastava D. (2015). microRNAs as developmental regulators. *Cold Spring Harb. Perspect. Biol.* 7, a008144 10.1101/cshperspect.a008144 26134312 PMC4484971

[BIO044800C13] Jeong I.-K., Oh D. H., Park S.-J., Kang J.-H., Kim S., Lee M.-S., Kim M.-J., Hwang Y.-C., Ahn K. J., Chung H.-Y. et al. (2011). Inhibition of NF-kappaB prevents high glucose-induced proliferation and plasminogen activator inhibitor-1 expression in vascular smooth muscle cells. *Exp. Mol. Med.* 43, 684-692. 10.3858/emm.2011.43.12.079 21975282 PMC3256296

[BIO044800C14] Jevrić M., Matić I. Z., Krivokuća A., Đorđić Crnogorac M., Besu I., Damjanović A., Branković-Magić M., Milovanović Z., Gavrilović D., Susnjar S. et al. (2019). Association of uPA and PAI-1 tumor levels and 4G/5G variants of PAI-1 gene with disease outcome in luminal HER2-negative node-negative breast cancer patients treated with adjuvant endocrine therapy. *BMC Cancer* 19, 71 10.1186/s12885-018-5255-z 30646864 PMC6332605

[BIO044800C15] Jin Y., Liang Z.-Y., Zhou W.-X. and Zhou L. (2019). Plasminogen activator inhibitor 2 (PAI2) inhibits invasive potential of hepatocellular carcinoma cells in vitro via uPA- and RB/E2F1-related mechanisms. *Hepatol. Int.* 13, 180-189. 10.1007/s12072-018-9920-8 30600477

[BIO044800C16] Kammoun T., Ben Abdallah R., Chabchoub I., Bahloul S., Aloulou H., Mahfoudh A. and Hachicha M. (2007). Hepatopulmonary syndrome and portal hypertension. *Tunis. Med.* 85, 170-173. 17665669

[BIO044800C17] Lin X., Lin B.-W., Chen X.-L., Zhang B.-L., Xiao X.-J., Shi J.-S., Lin J.-D. and Chen X. (2017). PAI-1/PIAS3/Stat3/miR-34a forms a positive feedback loop to promote EMT-mediated metastasis through Stat3 signaling in Non-small cell lung cancer. *Biochem. Biophys. Res. Commun.* 493, 1464-1470. 10.1016/j.bbrc.2017.10.014 28988111

[BIO044800C18] Liu C., Chen L., Zeng J., Cui J., Ning J.-N., Wang G.-S., Belguise K., Wang X., Qian G.-S., Lu K.-Z. and et al. (2015). Bone morphogenic protein-2 regulates the myogenic differentiation of PMVECs in CBDL rat serum-induced pulmonary microvascular remodeling. *Exp. Cell Res.* 336, 109-118. 10.1016/j.yexcr.2015.05.025 26071935

[BIO044800C19] Liu C., Gao J., Chen B., Chen L., Belguise K., Yu W., Lu K., Wang X. and Yi B. (2017). Cyclooxygenase-2 promotes pulmonary intravascular macrophage accumulation by exacerbating BMP signaling in rat experimental hepatopulmonary syndrome. *Biochem. Pharmacol.* 138, 205-215. 10.1016/j.bcp.2017.06.117 28642034

[BIO044800C20] Makarova A. M., Lebedeva T. V., Nassar T., Higazi A. A.-R., Xue J., Carinato M. E., Bdeir K., Cines D. B. and Stepanova V. (2011). Urokinase-type plasminogen activator (uPA) induces pulmonary microvascular endothelial permeability through low density lipoprotein receptor-related protein (LRP)-dependent activation of endothelial nitric-oxide synthase. *J. Biol. Chem.* 286, 23044-23053. 10.1074/jbc.M110.210195 21540184 PMC3123072

[BIO044800C21] Miyamoto A., Katsuta Y., Zhang X.-J., Li H.-L., Ohsuga M., Komeichi H., Shimizu S., Akimoto T. and Mizuno K. (2010). Effect of chronic methylene blue administration on hypoxemia in rats with common bile duct ligation. *Hepatol. Res.* 40, 622-632. 10.1111/j.1872-034X.2010.00640.x 20412326

[BIO044800C22] Muti P., Sacconi A., Hossain A., Donzelli S., Ben Moshe N. B., Ganci F., Sieri S., Krogh V., Berrino F., Biagioni F. et al. (2014). Downregulation of microRNAs 145-3p and 145-5p is a long-term predictor of postmenopausal breast cancer risk: the ORDET prospective study. *Cancer Epidemiol. Biomarkers Prev.* 23, 2471-2481. 10.1158/1055-9965.EPI-14-0398 25073626

[BIO044800C23] Patel N., Tahara S. M., Malik P. and Kalra V. K. (2011). Involvement of miR-30c and miR-301a in immediate induction of plasminogen activator inhibitor-1 by placental growth factor in human pulmonary endothelial cells. *Biochem. J.* 434, 473-482. 10.1042/BJ20101585 21175428 PMC3078570

[BIO044800C24] Pérez-Cremades D., Mompeón A., Vidal-Gómez X., Hermenegildo C. and Novella S. (2018). Role of miRNA in the regulatory mechanisms of estrogens in cardiovascular ageing. *Oxid. Med. Cell. Longev.* 2018, 6082387 10.1155/2018/6082387 30671171 PMC6317101

[BIO044800C25] Pusina S. (2018). Correlation of serum levels of urokinase Activation Plasminogen (uPA) and its inhibitor (PAI-1) with hormonal and HER-2 status in the early invasive breast cancer. *Med. Arch.* 72, 335-340. 10.5455/medarh.2018.72.335-340 30524164 PMC6282918

[BIO044800C26] Rajasekaran S., Pattarayan D., Rajaguru P., Sudhakar Gandhi P. S. and Thimmulappa R. K. (2016). MicroRNA regulation of acute lung injury and acute respiratory distress syndrome. *J. Cell. Physiol.* 231, 2097-2106. 10.1002/jcp.25316 26790856

[BIO044800C27] Shukla G. C., Singh J. and Barik S. (2011). MicroRNAs: processing, maturation, target recognition and regulatory functions. *Mol. Cell. Pharmacol.* 3, 83-92. 22468167 PMC3315687

[BIO044800C28] Swanson K. L., Wiesner R. H. and Krowka M. J. (2005). Natural history of hepatopulmonary syndrome: impact of liver transplantation. *Hepatology* 41, 1122-1129. 10.1002/hep.20658 15828054

[BIO044800C29] Wang L., Zhuang L., Rong H., Guo Y., Ling X., Wang R., Yu X. and Zhang W. (2015). MicroRNA-101 inhibits proliferation of pulmonary microvascular endothelial cells in a rat model of hepatopulmonary syndrome by targeting the JAK2/STAT3 signaling pathway. *Mol. Med. Rep.* 12, 8261-8267. 10.3892/mmr.2015.4471 26498873

[BIO044800C30] Wang X., Liu C., Wang J., Fan Y., Wang Z. and Wang Y. (2017). Oxymatrine inhibits the migration of human colorectal carcinoma RKO cells via inhibition of PAI-1 and the TGF-beta1/Smad signaling pathway. *Oncol. Rep.* 37, 747-753. 10.3892/or.2016.5292 27959430 PMC5355745

[BIO044800C31] Xu D., Gu J.-T., Yi B., Chen L., Wang G.-S., Qian G.-S. and Lu K.-Z. (2015). Requirement of miR-9-dependent regulation of Myocd in PASMCs phenotypic modulation and proliferation induced by hepatopulmonary syndrome rat serum. *J. Cell. Mol. Med.* 19, 2453-2461. 10.1111/jcmm.12631 26147104 PMC4594686

[BIO044800C32] Yang C., Lv K., Chen B., Yang Y., Ai X., Yu H., Yi B. and Lu K. (2018). miR144-3p inhibits PMVECs excessive proliferation in angiogenesis of hepatopulmonary syndrome via Tie2. *Exp. Cell Res.* 365, 24-32. 10.1016/j.yexcr.2018.02.009 29453975

[BIO044800C33] Yi B., Zeng J., Wang G., Qian G. and Lu K. (2013). Annexin A1 protein regulates the expression of PMVEC cytoskeletal proteins in CBDL rat serum-induced pulmonary microvascular remodeling. *J. Transl. Med.* 11, 98 10.1186/1479-5876-11-98 23587191 PMC3641942

[BIO044800C34] Yi B., Chen L., Zeng J., Cui J., Wang G., Qian G., Belguise K., Wang X. and Lu K. (2015). Ezrin regulating the cytoskeleton remodeling is required for hypoxia-induced myofibroblast proliferation and migration. *Front. Cardiovasc. Med.* 2, 10 10.3389/fcvm.2015.00010 26664882 PMC4671398

[BIO044800C35] Yuan X. and Liu N. (2011). Pioglitazone suppresses advanced glycation end product-induced expression of plasminogen activator inhibitor-1 in vascular smooth muscle cells. *J. Genet. Genomics* 38, 193-200. 10.1016/j.jgg.2011.04.001 21621740

[BIO044800C36] Zhang Y., Pan Y. and Xie C. (2018). miR-34a exerts as a key regulator in the dedifferentiation of osteosarcoma via PAI-1-Sox2 axis. *Cell Death Dis.* 9, 777 10.1038/s41419-018-0778-4 29991717 PMC6039486

