# Supplementary material for: MiR145-5p inhibits proliferation of PMVECs via PAI-1 in experimental hepatopulmonary syndrome rat pulmonary microvascular hyperplasia
Source: Biol Open. 2019 Nov 4;8(11):bio044800. doi: 10.1242/bio.044800 (PMC6899039; doi:10.1242/bio.044800)
Supplement: Supplementary information [file biolopen-8-044800-s1.pdf]

Table S1. Primers for this study.

Click here to Download Table S1

Table S2. Primers for this study.

Click here to Download Table S2

Table S3. Predicted microRNA binding site on PAI-1.

Click here to Download Table S3

Biology Open • Supplementary information
